# Electrical Transport, Structural, Optical and Thermal Properties of [(1−*x*)Succinonitrile: *x*PEO]-LiTFSI-Co(bpy)_3_(TFSI)_2_-Co(bpy)_3_(TFSI)_3_ Solid Redox Mediators

**DOI:** 10.3390/polym14091870

**Published:** 2022-05-03

**Authors:** Ravindra Kumar Gupta, Hamid Shaikh, Ahamad Imran, Idriss Bedja, Abrar Fahad Ajaj, Abdullah Saleh Aldwayyan

**Affiliations:** 1 King Abdullah Institute for Nanotechnology, King Saud University, Riyadh 11451, Saudi Arabia; aimran@ksu.edu.sa; 2 SABIC Polymer Research Center, College of Engineering, King Saud University, Riyadh 11421, Saudi Arabia; hamshaikh@ksu.edu.sa; 3 Cornea Research Chair, Department of Optometry, College of Applied Medical Sciences, King Saud University, Riyadh 11433, Saudi Arabia; bedja@ksu.edu.sa; 4 Department of Physics and Astronomy, College of Science, King Saud University, Riyadh 11451, Saudi Arabia; abrar.fa.ksa@gmail.com (A.F.A.); dwayyan@ksu.edu.sa (A.S.A.); 5 K.A. CARE Energy Research and Innovation Center, King Saud University, Riyadh 11451, Saudi Arabia

**Keywords:** dye-sensitized solar cells, redox mediator, solid polymer electrolytes, succinonitrile, electrical conductivity

## Abstract

The solar cell has been considered one of the safest modes for electricity generation. In a dye-sensitized solar cell, a commonly used iodide/triiodide redox mediator inhibits back-electron transfer reactions, regenerates dyes, and reduces triiodide into iodide. The use of iodide/triiodide redox, however, imposes several problems and hence needs to be replaced by alternative redox. This paper reports the first Co^2+^/Co^3+^ solid redox mediators, prepared using [(1−*x*)succinonitrile: *x*PEO] as a matrix and LiTFSI, Co(bpy)_3_(TFSI)_2_, and Co(bpy)_3_(TFSI)_3_ as sources of ions. The electrolytes are referred to as SN_E (*x* = 0), Blend 1_E (*x* = 0.5 with the ethereal oxygen of the PEO-to-lithium ion molar ratio (EO/Li^+^) of 113), Blend 2_E (*x* = 0.5; EO/Li^+^ = 226), and PEO_E (*x* = 1; EO/Li^+^ = 226), which achieved electrical conductivity of 2.1 × 10^−3^, 4.3 × 10^−4^, 7.2 × 10^−4^, and 9.7 × 10^−7^ S cm^−1^, respectively at 25 °C. Only the blend-based polymer electrolytes exhibited the Vogel-Tamman-Fulcher-type behavior (vitreous nature) with a required low pseudo-activation energy (0.05 eV), thermal stability up to 125 °C, and transparency in UV-A, visible, and near-infrared regions. FT-IR spectroscopy demonstrated the interaction between salt and matrix in the following order: SN_E < Blend 2_E < Blend 1_E << PEO_E. The results were compared with those of acetonitrile-based liquid electrolyte, ACN_E.

## 1. Introduction

Efficient utilization of fossil-fuel-based energy sources is one of the key factors of human social and economic development. However, this has led to an increase in the levels of greenhouse gases and pollution. The nuclear energy source is also not safe because of the hazards associated with it. Owing to the abundance of sunlight, the photovoltaic cell has emerged as an energy source, especially in regions near to and between the Tropics of Cancer and Capricorn (sunlight irradiance ~2 MWh m^−2^) [1]. 

One of the third generation photovoltaic cells, the dye-sensitized solar cell (DSSC), is highly attractive due to several advantages of DSSCs over other solar cells [2,3,4,5,6,7]. Some of the advantages are the simple cell structure, they are flexible and lightweight, the absence of toxic and less-available elements; energy payback time is less than a year, their all-direction-capturing of incident light; and their performance under real indoor and outdoor conditions. The first DSSC was reported by O’Regan and Gratzel in 1991 [8] with a power conversion efficiency (η) of 7.1% at 75 mW cm^−2^. They used a liquid electrolyte: tetrapropylammonium iodide, KI, and I_2_ in ethylene carbonate and acetonitrile (ACN). This electrolyte provided fast transport of the $I^{-}/I_{3}^{-}$ redox couple, (i) to regenerate dyes via the oxidation of I^−^ into $I_{3}^{-}$ at the mesoporous and nanostructured TiO_2_ working electrode, (ii) to reduce $I_{3}^{-}$ into I^−^ at the platinum counter electrode, and (iii) to inhibit back-electron transfer reactions. Since then, several $I^{-}/I_{3}^{-}$ redox mediators in a form of liquid, gel, or solid have been synthesized [9,10,11,12,13,14,15,16,17,18,19,20,21]. The DSSC has also been commercialized with the highest η-value of 11.9% at 100 mW cm^−2^ (1 sun), utilizing a liquid electrolyte: dimethyl-propyl imidazolium iodide (an ionic liquid), I_2_, LiI, and 4-*tert*-butylpyridine (TBP) in ACN [7,22]. The ionic liquid helped to reduce the organic solvent-related problems, thereby improving the stability of the device, though this required a hermetic sealing. 

Owing to their corrosive nature, dissolving of many of the commonly used sealants and metal interconnects, sublimation, and partial absorption of visible light around 430 nm of the iodine-based $I^{-}/I_{3}^{-}$ redox-couple, the researchers started to think of replacing this by one using a molecular species of similar type such as Br^−^/Br_2_ redox (e.g., Br_2_ and LiBr in ACN), one using metal complex-based redox such as Co^2+^/Co^3+^, and one using organic radicals, such as TEMPO [2,6,9,10,23,24,25,26,27,28,29]. The Co^2+^/Co^3+^ redox electrolytes, in general, with ACN as an organic solvent showed η of more than 10% at 1 sun for several dyes. For example, 11.9% for YD2-o-C8 dye [23], 12.3% for YD2-o-C8+Y123 dyes [23], 10.3% for JF419 dye [30], 13% for SM315 dye [24], 10.6% for Y123 dye [31], 11.4% for YD2-o-C8 dye [32], 10.2% for C101 dye [33], 10.5% for LEG4+D35+Dyenamo Blue dyes [34], 10.42% for FW1+WS5 dyes [35], 12.8% for SM342+Y123 dyes [36], 11% for AQ310 dye [37], 13.6% for ZL003 dye [38], 10.3% for H2 dye [39], and 11.2% for YD2-o-C8 dye [40]. 

The researchers used ionic liquid to suppress the organic solvent-related problems. Xu et al. [41] synthesized a compound [Co{3,3′-(2,2′-bipyridine-4,4′-diyl-bis(methylene)) bis(1-methyl-1H-imidazol-3-ium) hexafluorophosphate}_3_]^2+/3+^ and mixed with 1-propyl-3-methylimidazolium iodine, 1-ethyl-3-methyl imidazolium thiocyanate, guanidinium thiocyanate (GuSCN), and TBP. They reported η~7.37% at 1 sun for N719 dye. Kakiage et al. reported η~12.5% at 1 sun for ADEKA-1 dye [42] and η~14.3% at 1 sun for ADEKA-1+LEG4 dyes [43], utilizing an electrolyte solution: [Co^2+^(phen)_3_](PF_6_)_2_, [Co^3+^(phen)_3_](PF_6_)_3_, LiClO_4_, NaClO_4_, tetrabutyl ammonium hexafluorophosphate, tetrabutylphosphonium hexafluorophosphate, 1-hexyl-3-methylimidazolium hexa fluorophosphate, TBP, 4-trimethylsilylpyridine, 4-methylpyridine, 4-cyano-4′-propyl biphenyl, 4-cyano-4′-pentylbiphenyl, 4-cyano-4′-octylbiphenyl in ACN. Wang et al. [44] reported η~8.1% at 1 sun for D205 dye with a redox mediator, bis(3-butyl-1-methylimidazolium) tetraisothiocyanato cobalt, 1-propyl-3-methyl-imidazolium iodine, nitrosyl tetrafluoroborate, LiClO_4_, and TBP in methoxy propionitrile. 

The researchers synthesized the Co^2+^/Co^3+^ redox mediators in a gel (quasi-solid) form as well. The gel was prepared by incorporating a large amount of organic solvent mixed with a redox couple into an inorganic or organic frame. So far nanoparticles of SiO_2_ (η~2.58% at 1 sun for D35 dye) [45], TiO_2_ (η~5.1% at 1 sun for N719 dye) [46], and TiC (η~6.29% at 1 sun for N719 dye) [46] were used to form an inorganic frame. An organic frame was prepared using poly(ethylene glycol) with gelatin (η~4.1% at 1 sun for MK2 dye) [47], bisphenol A ethoxylate dimethacrylate with poly(ethylene glycol) methyl ether methacrylate (η~6.4% at 1 sun for LEG4 dye) [48], poly(ethylene glycol)/poly(methyl methacrylate) (η~1.9% at 1 sun for N719 dye) [49], poly(vinylidene fluoride-co-hexafluoropropylene) (η~8.7% at 1 sun for MK2 dye [50]; η~4.34% at 1 sun for Z907 dye [51], η~7.1% at 1 sun for MK2 dye [52]), poly(ethylene oxide-co-2-(2-methoxyethoxy) ethyl glycidyl ether-co-allyl glycidyl ether) (η~3.59% at 1 sun for MK2 dye and η~1.74% at 1 sun for Z907 dye) [53], poly(ethylene oxide-co-2-(2-methoxyethoxy) ethyl glycidyl ether (η < 0.1% at 1 sun for L0 dye) [54], poly(ethylene oxide) (η~21.1% at 200 lx for Y123 dye) [55], poly(ethylene oxide)-poly(methyl methacrylate) blend (η~18.7% at 200 lx for Y123 dye) [55], hydroxypropyl cellulose (η~9.1% at 0.7 sun for N719 dye) [56], and hydroxyethyl cellulose (η~4.5% at 1 sun for N3 dye) [57]. 

Unfortunately, the liquid nature of electrolytes creates internal pressure in the DSSCs at the ambient temperature range (50–80 °C), resulting in a leakage of solvent, thereby requiring hermetic sealing [9,10,11,12,13,14,15,16,17]. This also makes the manufacturing of DSSCs non-scale-up. A gel electrolyte exhibits problems similar to those of a liquid electrolyte, hence, it needs to be replaced by a solid one to sustain it in the hot weather of Gulf countries. However, until now no Co^2+^/Co^3+^ redox mediator in solid form has been reported. 

Earlier, a high-molecular-weight poly(ethylene oxide) (PEO) was used as a polymer matrix of the I^−^/I_3_^−^ redox-based solid polymer electrolytes, PEO-PQ-MI-I_2_, where M represents an alkali metal cation [9,10,11,12,13,14,15,16,17]. The PEO offered its self-standing film-forming, thermal stability up to 200 °C, it was eco- and bio-benign, was of relatively low material cost, the dissociation/ complexation of salt due to its moderate dielectric constant (ε_25°C_)-value (5–8), Gutmann donor number of 22, had just the right spacing between coordinating ethereal oxygens for maximum solvation of the Li^+^ ions, and the segmental motion of polymeric chains for the ion transport through ethereal oxygen [58,59,60,61]. Ionic liquids, low molecular weight polymers, and copolymerization were used as plasticizers (PQs) to reduce PEO crystallinity (χ), thereby increasing the σ_25 °C_- and η-values. 

Gupta et al. [62,63,64,65,66,67,68] showed that the equal weight proportion of succinonitrile (SN) can be used as a plasticizer without hampering the thin-film forming property of the PEO. This blending resulted in several beneficial properties, such as a higher σ_25°C_-value, ~10^−8^ S cm^−1^ than the PEO (σ_25°C_ ~10^−10^ S cm^−1^), a lower χ-value, ~25% than the PEO (~82%), and higher thermal stability up to ~125 °C than the SN (~75 °C) [62]. The PEO-SN-MI-I_2_ solid polymer electrolytes achieved σ_25°C_-value 3–7 × 10^−4^ S cm^−1^, transparency more than 95% in visible and IR regions, χ~0%, thermal stability up to ~125 °C, and η-value between 2 and 3.7% at 1 sun with Ru-based N719 dye. The solid solvent/ plasticizing property of the plastic crystal, SN is due to its low molecular weight, high molecular diffusivity at the plastic phase between −35 °C (crystal-to-plastic-crystal phase transition temperature, *T*_pc_) and 58 °C (melting temperature, *T*_m_), low *T*_m_-value, high ε-value ~55 at 25 °C and 62.6 at 58 °C, nitrile group for ion transport, and waxy nature [69,70,71,72,73]. 

**Figure 1:**
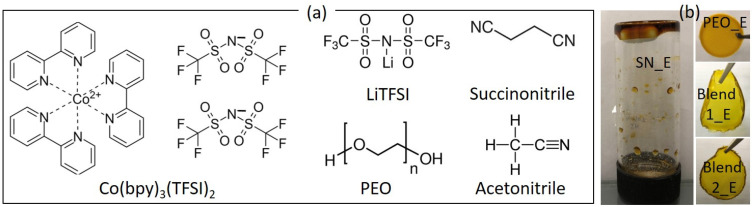
(**a**) Chemical structure of ingredients. (**b**) Optical image of solid redox mediators.

In this work, we have extended the concept of blending for achieving the high electrical conductivity of the Co^2+^/Co^3+^ solid redox mediators. We reported electrical, structural, optical, and thermal properties of new [(1−*x*)SN: *x*PEO]-LiTFSI-Co(bpy)_3_(TFSI)_2_-Co(bpy)_3_(TFSI)_3_ solid redox mediators. The composition, *x* is 0, 0.5, and 1 in weight fraction. Other notations, bpy and TFSI stand for tris-(2,2′-bipyridine) and bis(trifluoromethyl) sulfonylimide, respectively. These solid redox mediators are based on a liquid electrolyte (0.1-M LiTFSI, 0.25-M Co(bpy)_3_(TFSI)_2_, and 0.06-M Co(bpy)_3_(TFSI)_3_ in acetonitrile), which resulted in η of 13% with SM315 dye [24]. This liquid electrolyte is hereafter referred to as ACN_E. We just replaced acetonitrile with succinonitrile for synthesizing SN_E (*x* = 0). Succinonitrile was then replaced by PEO for synthesizing PEO_E (*x* = 1 in weight fraction). This had the ethereal oxygen of the PEO-to-lithium ion mole ratio, abbreviated as EO/Li^+^ of 226. We also used a blend containing SN and PEO in an equal weight fraction for retaining the beneficial properties of SN and PEO, as discussed earlier. The value of EO/Li^+^ was kept at either 113 (Blend 1_E) or 226 (Blend 2_E) for understanding its effect on the electrical transport properties [65]. Figure 1a shows the chemical structure of the ingredients. The solid nature of SN_E, PEO_E, Blend 1_E, and Blend 2_E is shown in Figure 1b. Ionic salts with TFSI^−^ anion were used because TFSI^−^ offers a low value of lattice energy with delocalized electrons, making the salt highly dissociable in the solvent with a less anionic contribution to the total conductivity [58,59,60,61]. The lithium salt is thermally and electrochemically stable as well [58,59,60,61,74]. Owing to the small size, the Li^+^ ions get intercalated on the TiO_2_ nanoparticles of the DSSC, leading to faster electron injection from the excited dye molecules to the conduction band of the TiO_2_, thereby the higher photocurrent [67,75]. Contrary to this, the cobalt ions adsorb on the skirt of the TiO_2_ nanoparticles, resulting in a negative shift of the Fermi level of the TiO_2_ nanoparticles, thereby resulting in the higher open-circuit voltage. It is also known that an ion with a large size acts as a plasticizer in a polymer electrolyte, resulting in higher electrical conductivity [65,67,75].

We determined σ-value at different temperatures for knowing the nature of the electrolyte and determining the activation energy. The electrical transport properties were elucidated by X-ray diffractometry (XRD), Fourier-transform infrared (FT-IR) spectroscopy, UV-visible spectroscopy, polarized optical microscopy (POM), and differential scanning calorimetry (DSC). We used thermogravimetric analysis (TGA) for the thermal stability study. 

## 2. Materials and Methods

### 2.1. Materials

Succinonitrile, PEO (1-M g mol^−1^), and LiTFSI were procured from Sigma Aldrich, Inc., St. Louis, MO, USA. Cobalt salts, Co(bpy)_3_(TFSI)_2_ (DN-C13) and Co(bpy)_3_(TFSI)_3_ (DN-C14) were procured from Dyenamo AB, Stockholm, Sweden. These chemicals were used without purification.

### 2.2. Synthesis

Table 1 shows the composition of ACN_E, SN_E, PEO_E, Blend 1_E, and Blend 2_E. As per the procedure of Mathew et al. [24], ACN_E was synthesized using LiTFSI (0.1 M), DN-C13 (0.25 M), and DN-C14 (0.06 M) in acetonitrile under stirring at 65 °C for 24 h. The SN_E was prepared identically by dissolving the salts in succinonitrile. The PEO_E and Blends 1_E & 2_E were prepared using the solution cast method. The ingredients were dissolved in 20-mL acetonitrile by vigorous stirring at 65 °C for 48 h. This resulted in a homogeneous polymeric solution which was poured on a Teflon Petri dish followed by drying at room temperature in a nitrogen gas atmosphere for two weeks and in a vacuum desiccator for a day. This produced a self-standing film of the solid polymer electrolyte.

### 2.3. Characterizations

A specific sample holder [72] was used to measure the electrical conductivity of the ACN_E and SN_E electrolytes. The liquid electrolyte was poured on a space (area, *A* ~0.16 cm^−2^ and thickness, *l*~0.05 cm) created by a Teflon spacer between platinum plates (blocking electrode). For determining the electrical conductivity of the PEO_E and Blends 1_E & 2_E solid polymer electrolytes, another sample holder [64], having stainless steel plate as a blocking electrode, was used. The sandwiched electrolyte was subjected to 20 mV ac voltage and monitoring of real and imaginary impedances from 100 kHz to 1 Hz by a Palmsens4 impedance analyzer (PalmSens BV, Houten, the Netherlands). This resulted in a Nyquist curve, thereby a bulk resistance (*R*_b_) and then the electrical conductivity (σ) using the formula, σ = *l*/(*A R*_b_) [76]. 

For the XRD pattern of the solid electrolyte film, a D2 Phaser Bruker x-ray diffractometer (Karlsruhe, Germany) was used. The pattern was collected using the CuKα radiation (1.54184 Å) in a range of 10–40° with a step of 0.06°. The FT-IR spectrum of the electrolyte film on a potassium bromide pellet was recorded in a range of 400–4000 cm^−1^ and a resolution of 1 cm^−1^ using a Spectrum 100 Perkin Elmer FT-IR spectrometer (Waltham, MA, USA). The spectrum was analyzed using EZ-OMNIC software, ver. 7.2a (Thermo Scientific Inc., Waltham, MA, USA). 

Transmittance spectrum of the electrolyte film (thickness 2–3 µm) was collected using an Agilent UV-visible spectrometer (model 8453, Santa Clara, CA, USA). The POM image with a magnification of 100× for the polymer electrolyte film (thickness 2–3 µm) was obtained using a computer interfaced ZZCAT polarized optical microscope (Zhuzhou, Hunan, China). 

The DSC curve of the electrolyte was measured using a DSC-60A differential scanning calorimeter (Shimadzu, Kyoto, Japan) under the purging of nitrogen gas with 10 °C min^−1^ heating rate and in the range of −50 to 90 °C. For the TGA curve, the weight loss of the electrolyte was monitored using a Shimadzu DTG-60H unit in the temperature range of room temperature to 550 °C with a heating rate of 10 °C min^−1^ under the purging of nitrogen gas. 

## 3. Results

### 3.1. Electrical Transport Properties

**Figure 2:**
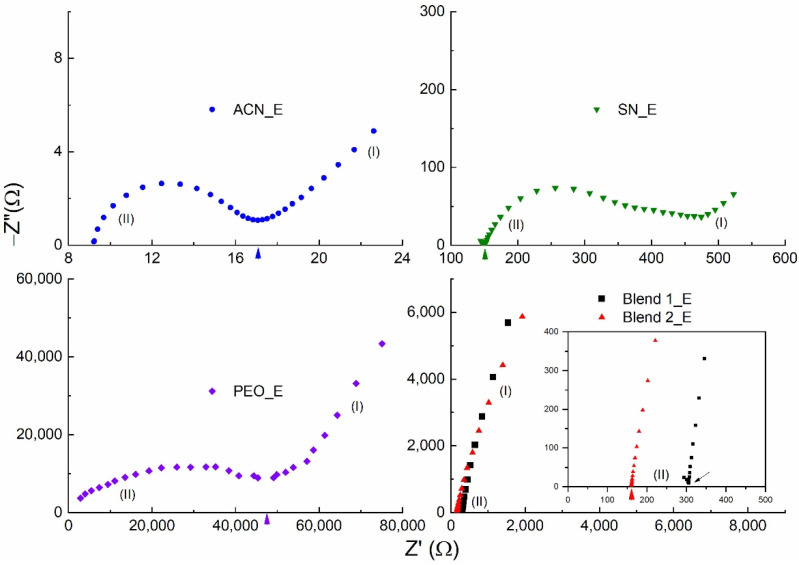
Nyquist curves of the solid (SN_E, PEO_E, Blend 1_E, and Blend 2_E) and liquid (ACN_E) redox mediators at 25 °C. (I) and (II) represent low- and high-frequency domains, respectively. The inset shows the high-frequency domain of the Blends 1_E and 2_E.

Figure 2 shows the Nyquist curves for the liquid (ACN_E) and solid (SN_E, PEO_E, Blend 1_E, and Blend 2_E) redox mediators at 25 °C. These curves portrayed (i) the blocking electrode effect in the low-frequency domain, and (ii) the ionic diffusion effect in the high-frequency domain [76]. Being a liquid electrolyte, ACN_E depicted a perfect semi-circle in the high-frequency domain. SN_E and PEO_E also had a semi-circle; however, the semi-circle was slightly and largely depressed for the former and latter, respectively. This is most probably due to the existence of the plastic crystalline phase of succinonitrile and the semi-crystalline phase of PEO, respectively [72,76]. Contrary to this, Blends 1_E and 2_E had no semi-circle, indicating the existence of amorphous domains, the semi-random motion of short polymer chains, and the segmental motion, demonstrating the plasticizing effect of the succinonitrile [58,59,60,61,64,65,77,78]. The bulk resistance is marked by an arrow in the Nyquist curve and is used to calculate the σ_25°C_-value of the electrolyte. 

**Figure 3:**
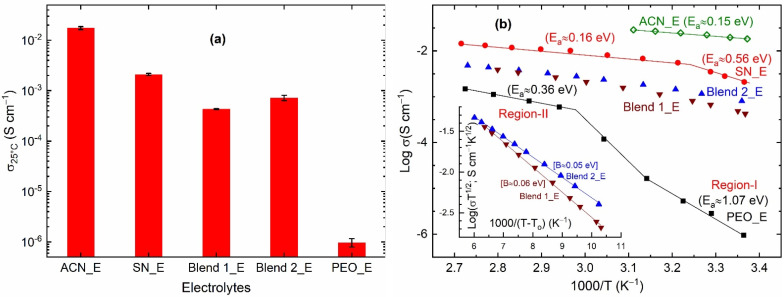
(**a**) Electrical conductivity (σ_25°C_) and (**b**) log σ vs. *T*^−1^ plots of the solid redox mediators, SN_E, PEO_E, Blend 1_E, and Blend 2_E. Inset in (**b**) is VTF plots of Blends 1_E and 2_E. ACN_E, liquid electrolyte.

Figure 3a shows electrical conductivity σ_25°C_) of solid electrolytes, SN_E, Blend 1_E, Blend 2_E, and PEO_E along with that of the liquid electrolyte, ACN_E. The ACN_E exhibited σ_25°C_~1.7 × 10^−2^ S cm^−1^, which is similar to those reported earlier for liquid electrolytes [60]. The high electrical conductivity is due to ε_25°C_ of 36.6, donor number of 14.1 kcal mol^−1^, molar enthalpy of 40.6 kJ mol^−1^, and acceptor number of 18.9, helping to dissolve the salt completely and solvate the ions easily [79,80]. The replacement of ACN by SN resulted in SN_E with the σ_25°C_-value less than an order of magnitude to ~2.1 × 10^−3^ S cm^−1^. This conductivity value is similar to those obtained earlier for the SN-LiTFSI [69] and SN-LiI-I_2_ [72] electrolytes. As discussed earlier [69,72], this is due to the solid solvent property of the succinonitrile. The replacement of SN by PEO resulted in PEO_E with the σ_25°C_-value of ~9.7 × 10^−7^ S cm^−1^, which is 3-orders of magnitude less. This is legitimate too. The pure PEO-based solid polymer electrolytes are known to have high PEO crystallinity, hindering ion transport [58,59,60,61,64,65]. The blend-based solid polymer electrolytes, however, showed σ_25°C_-value less than that of SN_E and higher than that of PEO_E. Blend 1_E and Blend 2_E exhibited σ_25°C_ of ~4.3 × 10^−4^ and ~7.2 × 10^−4^ S cm^−1^, respectively. As observed earlier [62,63,64,65,66,67,68], this is due to the plasticizing property of the succinonitrile. Also, a competition between the nitrile group of succinonitrile and the ethereal oxygen of PEO to bind metal ions leads to more free ions for transport [64,65]. Besides, the availability of a huge number of large-sized TFSI^−^ ions is helpful to produce more amorphous regions in the Blends 1_E and 2_E for easy ion transport [74]. One can expect a similar scenario for Co^2+^/Co^3+^ ions too [65,75]. It is also notable that Blend 2_E had higher electrical conductivity than Blend 1_E. This is due to more amorphous regions for ion transport in the Blend 2_E as demonstrated by the FT-IR spectroscopy, UV-visible spectroscopy, and DSC studies, which will be discussed later. 

Figure 3b shows log σ vs. *T*^−1^ plots of the solid (SN_E, Blend 1_E, Blend 2_E, and PEO_E) and liquid (ACN_E) redox mediators. The ACN_E, SN_E, and PEO_E portrayed a linear curve, revealing the thermally activated Arrhenius-type behavior of molecules/ polymeric chains. Blends 1_E and 2_E depicted a slightly downward curve, indicating the existence of an amorphous phase, which follows the Vogel-Tamman-Fulcher (VTF)-type behavior. We have observed these trends for several $I^{-}/I_{3}^{-}$ redox mediators [64,65,68,72,73]. The Arrhenius behavior is expressed by the equation, σ = σ_o_ exp[−*E*_a_/k_B_*T*], where σ_o_ is the pre-exponential factor, *E*_a_ is the activation energy, and k_B_ is the Boltzmann constant. While the VTF behavior is represented by an expression, σ = *AT*^−1/2^ exp[−*B*/k_B_(*T* − *T*_o_)], where *A* is the pre-exponential factor, *B* is the pseudo-activation energy, and *T*_o_ is the temperature at which the free volume vanishes. The *E*_a_-value calculated from the slope of the Arrhenius plot is as follows: 0.56 eV (Region-I) and 0.16 eV (Region-II) for SN_E; and 1.07 eV (Region-I) and 0.36 eV (Region-II) for PEO_E. Region-I represents the solid-state region for SN_E and PEO_E, while Region-II corresponds to the liquid state for SN_E and the amorphous phase for PEO_E. The activation energy for SN_E in Region-II is similar to that observed (0.15 eV) for the liquid electrolyte, ACN_E. The pseudo-activation energy (*B*) calculated from the slope of the VTF plot is as follows: 0.06 eV and 0.05 eV for Blends 1_E and 2_E, respectively. The low activation energy values for the Blends 1_E and 2_E indicate easy ion transport, which is required for the DSSC application. 

### 3.2. Structural Properties

**Figure 4:**
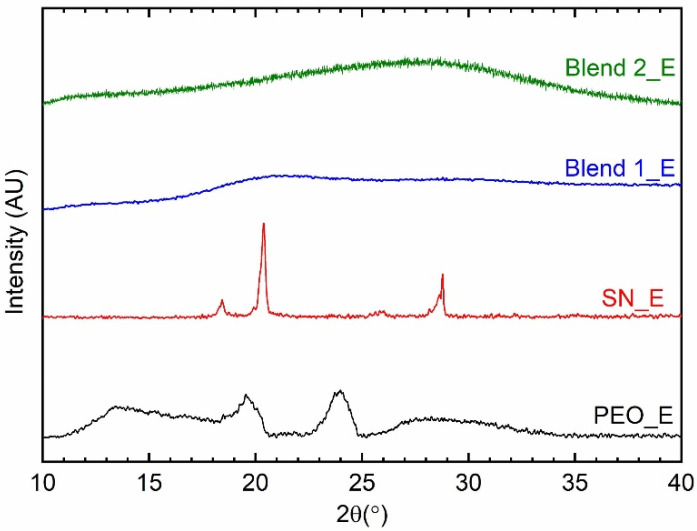
XRD patterns of the solid redox mediators, SN_E, PEO_E, Blend 1_E, and Blend 2_E.

Figure 4 shows XRD patterns of the solid redox mediators, SN_E, PEO_E, Blend 1_E, and Blend 2_E. The SN_E and PEO_E exhibited characteristic reflection peaks of succinonitrile and poly(ethylene oxide), respectively, though their peaks are broader and weaker than those of pure matrices, succinonitrile, and PEO. These indicate molecular disorder for SN_E and an increase in amorphicity for PEO_E [64,65,72], resulting in significantly enhanced electrical conductivity as compared to the pure matrices. The available cations and anions, having a large size, also acted as plasticizers and contributed to increasing the amorphicity [67,72,73,74,75]. Also, these electrolytes did not show any peak corresponding to cobalt and lithium salts, indicating complete salt dissociation/ complexation. The Blends 1_E and 2_E portrayed the absence of reflection peaks of ingredients, revealing the arrest of the glassy phase. As mentioned earlier, succinonitrile is a very good plasticizer to decrease the crystallinity of PEO [63,64,65]. This is also accompanied by the PEO-SN blend matrix-metal ions interaction, where SN molecules are more active [68,77,78]. These results are also supported by the findings of the FT-IR spectroscopy, which are discussed below. 

**Figure 5:**
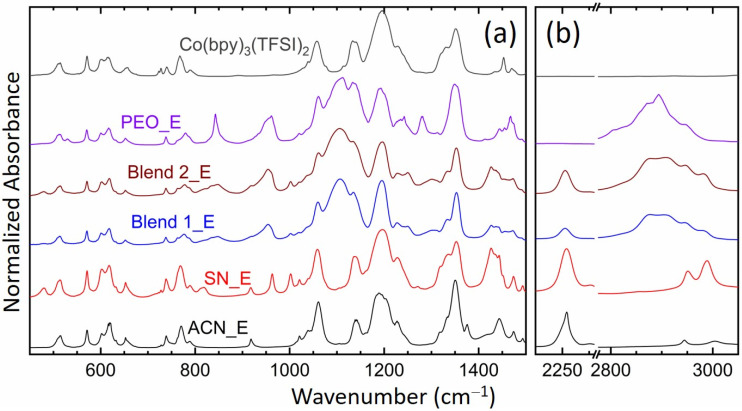
FT-IR spectra of the solid (SN_E, PEO_E, Blend 1_E, and Blend 2_E) and liquid (ACN_E) redox mediators. The spectrum of the Co(II) salt is also included for direct comparison. (**a**) Fingerprint region. (**b**) ν_C__≡N_ and ν_CH_ regions.

Figure 5 shows FT-IR spectra of the solid redox mediators, SN_E, PEO_E, Blend 1_E, and Blend 2_E. This figure also shows the spectra of liquid electrolyte, ACN_E and Co(bpy)_3_(TFSI)_2_ salt for comparison. The spectrum of the Co(bpy)_3_(TFSI)_3_ salt (data not shown) was similar to that of the Co(bpy)_3_(TFSI)_2_ salt. The observed vibrational frequencies of liquid and solid redox mediators along with those of constituents, ACN [81], SN [82], PEO [83], PEO-SN blend [62,64,65], LiTFSI [74,84,85], and Co(bpy)_3_(TFSI)_2_ [84,85,86], are listed in Table 2. This also shows the corresponding assignments. It is worth mentioning that the PEO-SN blending occurs via the interaction of the ethereal oxygen with the nitrogen of SN [62,64]. Wen et al. [84] asserted that the vibrational peaks at 1057, 1133, 1196, and 1351 cm^−1^ correspond to free and unpaired anions that are strongly solvated. Rey et al. [85] showed that the vibrational peaks at 1229 and 1331 cm^−1^ correspond to the ion-pairing peaks, though these peaks present free ions too if their position did not get changed with increasing salt concentration. The formation of an ion pair reduces the number of free ions. However, being uncharged and having nearly the same size as the cation, it has somewhat higher mobility in the polar polymer/solvent and lower solvent-salt interaction for increased amorphicity [84]. These have been discussed below.

The solute-solvent interaction in ACN_E is quite low as indicated by the comparatively unaltered position of several modes of ACN and ionic salts. We observed a change only at 739 cm^−1^ (ν_a,C-CN_) and 2255 cm^−1^ (ν_s,C__≡N_) of the ACN_E relative to those of the ACN because of nitrile-metal ions coordination [87]. The ion-pairing peaks were present, however as weak shoulders only. This indicates the availability of a huge number of free ions for migration along with a negligible level of ion-pairing, resulting in a high value of σ_25°C_ for the ACN_E. In this electrolyte, the metal cations migrate through the nitrile group of the ACN [69]. The SN_E showed a scenario similar to ACN_E except at 769 (δ_CH2_, ring), 1228 (t_CH2_, i.p.ring), 1443 (δ_a,CH2_, ring), and 1474 cm^−1^ (δ_a,CH2_, ring), revealing the SN-ring interaction. Similar to ACN_E, SN_E portrayed a weak ion-pairing peak at 1228 cm^−1^. These indicate the availability of a large number of free ions for migration, however, with a higher level of ion-pairing, resulting in a lower σ_25°C_-value for SN_E than ACN_E. PEO_E experienced the PEO-salt interaction via shifts to 780 (767 cm^−1^, ring), 1113 (1109 cm^−1^, PEO; ν_a,COC_), 1134 (1149 cm^−1^, PEO; ν_CC_, ν_a,COC_), and 1349 (1342 cm^−1^, PEO; ω_a,CH2_, ν_a,SO2_). These were accompanied by a shift in ν_CH2_ modes of the PEO from 2861 to 2872 cm^−1^ and from 2889 to 2894 cm^−1^, indicating a decrease in C-H bond length for ion solvation, and thereby increasing the amorphicity of the electrolyte [64,65,74]. However, the increase in amorphicity was inadequate to sufficiently increase the electrical conductivity as suggested by the absence of the ion-paring peaks. The Blend 1_E and Blend 2_E observed no significant change in the position of several modes, except at 777 cm^−1^ (δ_CH2_, SN) and 1437 cm^−1^ (δ_CH2_, ring) corresponding to the SN-bpy ligand interaction, and at 2253 cm^−1^ (ν_s,C__≡N_, SN) for the interaction of the nitrile-metal ions [87]. The blend-based electrolytes portrayed ion-pairing peaks at 1227 and 1334 cm^−1^ as well as a blue shift in the stretching C-H modes of PEO in the region, 2800–3050 cm^−1^, indicating a conformational change to form the amorphous phase. The blue shift was higher for the Blend 2_E. These findings suggest that succinonitrile and cobalt salts are crucial for improving the amorphicity of the blend-based electrolyte, thereby, enhancing the electrical conductivity. 

**Figure 6:**
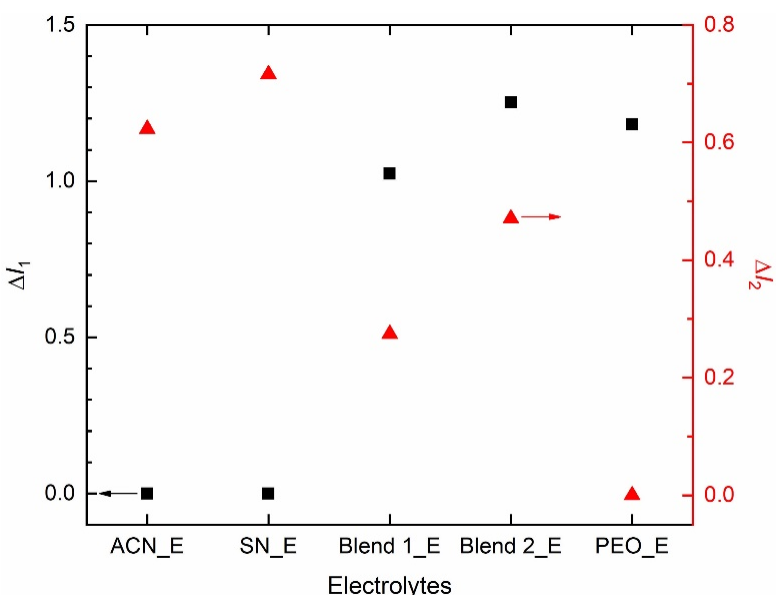
Relative intensities, Δ*I*_1_ and Δ*I*_2_ of the electrolytes. For definition, please see the text.

Figure 6 shows relative intensities, Δ*I*_1_ (=*I*(1105 cm^−1^; ν_s,COC_; PEO)/*I*(1196 cm^−1^; ν_a,CF3_; TFSI^−^) and Δ*I*_2_ (=*I*(2255 cm^−1^; ν_s,C__≡N_; acetonitrile or succinonitrile)/*I*(1196 cm^−1^; ν_a,CF3_; TFSI^−^) of the redox mediators. Intensity had the following order: ACN_E = SN_E (=0) << Blend 1_E = Blend 2_E = PEO_E (=1) at 1105 cm^−1^ and ACN_E < SN_E ≈ Blend 1_E > Blend 2_E < PEO_E at 1196 cm^−1^, resulting in Δ*I*_1_ as Blend 2_E > PEO_E > Blend 1_E >> SN_E = ACN_E (=0). This shows the effect of the PEO-salt interaction on the conformational change of PEO to form the amorphous phase, which is the highest for the Blend 2_E. A similar assertion can be made using Δ*I*_2_, which had the following order: SN_E > ACN_E > Blend 2_E > Blend 1_E >> PEO_E (=0). This demonstrates the effect of nitrile-salt interaction on the conformational change of ACN/SN to form the disordered/ amorphous structure, which is higher for SN_E than ACN_E, and more for Blend 2_E than Blend 1_E. These results are also supported by the UV-visible spectroscopy, POM, and DSC studies and are discussed in the later sections.

### 3.3. Optical Properties

**Figure 7:**
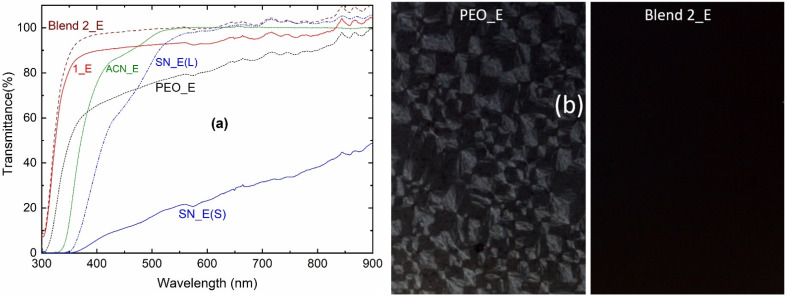
(**a**) Transmittance spectra of the ACN_E, SN_E (L, liquid; S, solid), PEO_E, Blend 1_E, and Blend 2_E. (**b**) polarized optical micrographs of the PEO_E and Blend 2_E.

Figure 7a shows the transmittance spectra of the solid redox mediators, SN_E, PEO_E, Blend 1_E, and Blend 2_E as well as liquid electrolytes, ACN_E and SN_E(L). In the UV-A region at 350 nm, the transmittance order was as follows: Blend 2_E (88.1%) > Blend 1_E (81.4%) >> PEO_E (49.6%) >> ACN-E (13.9%) >> SN_E(L) = SN_E(S) (=0%). In the visible region at 555 nm, transparency had the following order: ACN-E (99.9%) ≈ Blend 2_E (99.8%) > SN_E(L) (97%) > Blend 1_E (92.9%) > PEO_E (78.9%) >> SN_E(S) 21.6%. The Blend 2_E portrayed the transparency of all the UV-A, visible, and near-infrared regions; while ACN_E and SN_E(L) showed the transparency in the visible and near-infrared regions only. This shows the supremacy of Blend 2_E over ACN_E and SN_E(L) in terms of transparency. The high value of the transmittance for Blends 1_E and 2_E suggests a low level of the PEO crystallinity [62,65]. The transmittance of Blend 2_E is higher than Blend 1_E, revealing higher amorphicity for the Blend 2_E. These findings are also indicated by the POM study, which has been discussed below. 

Figure 7b shows polarized optical micrographs of the PEO_E and Blend 2_E. The Blend 1_E had a micrograph similar to the Blend 2_E. The PEO_E micrograph depicted two parts: (i) several diamond-like spherulites due to the short and randomly oriented PEO chains; and (ii) a little dark region due to the amorphous domain [64,65]. This indicates the presence of a highly crystalline phase of the PEO, resulting in low electrical conductivity. Blend 2_E showed a complete dark region indicating arrest of the amorphous phase, which is responsible for the higher electrical conductivity. These findings are also supported by the DSC study, which has been described below. 

### 3.4. Thermal Properties

**Figure 8:**
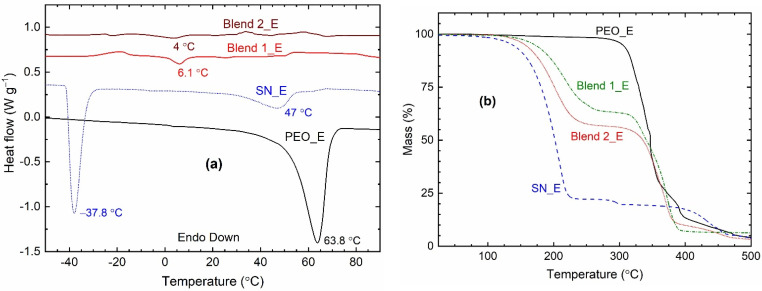
(**a**) DSC and (**b**) TGA curves of the solid redox mediators.

Figure 8a shows DSC curves of the solid redox mediators, SN_E, PEO_E, Blend 1_E, and Blend 2_E. The DSC curves showed endothermic peaks corresponding to the melting temperature of the electrolytes, which are as follows: ~63.8 °C for PEO_E, ~47 °C for SN_E, ~6 °C for Blend 1_E, and ~4 °C for Blend 2_E. These values are less than those of pure matrices: ~65.7 °C for PEO [62], ~57.7 °C for SN [72], and ~30.1 °C for PEO-SN blend [62]. This indicates a decrease in the crystallinity of the PEO and SN, which are responsible for the conductivity enhancement of the electrolytes [62,63,64,65,72]. It is worth mentioning that the PEO, thereby the related compounds, do not lose the thin film-forming property of the PEO even after the *T*_m_-value [58,59]. In fact, the electrolyte becomes amorphous, which provides highly conducting pathways for easy ion transport [62,63,64,65]. The TGA study discussed later showed that Blend-based electrolytes are thermally stable up to 125 °C. The SN_E depicted another endothermic peak at −37.8 °C, which is similar to that of pure SN (−38.4 °C [72]) and corresponds to the *T*_pc_. The Blends 1_E and 2_E did not show the *T*_pc_-peak. This is due to the matrix-salt interaction phenomenon [62,63,64,65]. It is also worth mentioning that the area under the melting point peak corresponds to the heat enthalpy of the electrolyte [62,63,64,65]. This area showed the following order for the PEO-based electrolytes: PEO_E ≫ Blend 1_E > Blend 2_E, indicating an extremely low level of crystallinity for the Blends 1_E and 2_E, which is one of the unique properties of the SN-PEO blend-based electrolytes [64,65,77,78]. 

Figure 8b shows TGA curves of the solid redox mediators, SN_E, PEO_E, Blend 1_E, and Blend 2_E. The thermal stability of the electrolyte is estimated by the initial plateau region for the mass, which is as follows: ~75 °C for SN_E, ~200 °C for PEO_E, and ~125 °C for Blends 1_E and 2_E. These values are similar to pure matrices reported earlier [62]. The SN_E and PEO_E exhibited a huge drop at ~125 °C and ~300 °C, respectively, due to single-stage decomposition. However, the Blends 1_E and 2_E portrayed two-stage degradation, first at ~125 °C and second at ~300 °C, corresponding to the decomposition of the ingredients, earlier SN, and later PEO.

## 4. Discussion

We synthesized solid redox mediators using [(1−*x*)SN: *x*PEO] as a solid matrix and LiTFSI, Co(bpy)_3_(TFSI)_2_, and Co(bpy)_3_(TFSI)_3_ ionic solids as sources of ions, following the procedure of Mathew et al. [24], which had acetonitrile as a solvent and 0.1-M LiTFSI, 0.25-M Co(bpy)_3_(TFSI)_2_, and 0.06-M Co(bpy)_3_(TFSI)_3_ as sources of ions. The acetonitrile-based liquid electrolyte, ACN_E exhibited σ_25__°C_ of ~1.7 × 10^−2^ S cm^−1^. The composition, *x* = 0 resulted in a pure plastic crystal-based electrolyte, SN_E with σ_25__°C_ of ~2.1 × 10^−3^ S cm^−1^. This value is similar to those of other succinonitrile-based electrolytes [69,72] and is attributed to the solid solvent property of the succinonitrile. The *x* = 1 yielded a pure PEO-based solid polymer electrolyte (EO/Li^+^ = 226), PEO_E with σ_25__°C_ of ~9.7 × 10^−7^ S cm^−1^. The PEO is a well-known polymer matrix for synthesizing a solid polymer electrolyte; however, the highly crystalline nature of the PEO results in poor electrical conductivity [58,59,60,61,64,65]. The blend-based solid polymer electrolytes (*x* = 0.5), Blend 1_E (EO/Li^+^ = 113) and Blend 2_E (EO/Li^+^ = 226) had σ_25__°C_ of ~4.3 × 10^−4^ and ~7.2 × 10^−4^ S cm^−1^, respectively, which were closer to that of the SN_E, disclosing the effect of plasticization property of succinonitrile [64,65]. 

The investigation of the temperature variation of electrical conductivity resulted in a log σ−*T*^−1^ plot, which was linear for ACN_E, SN_E, and PEO_E, and downward for Blend 1_E and Blend 2_E. The former corresponds to the thermally activated behavior of a homogeneous electrolyte. The latter corresponds to a mixed effect of amorphous domains, the semi-random motion of short polymer chains, and the segmental motion, which was produced by succinonitrile through the interaction with PEO [58,59,60,61,62,64,65,74,77,78]. The Arrhenius-type plot resulted in activation energy of 0.56 eV for SN_E and 1.07 eV for PEO_E in the solid-state region, more than the limiting condition (0.3 eV) for a device application [88]. The ACN_E had an activation energy of 0.15 eV. The VTF-type plot resulted in pseudo-activation energy of 0.06 eV for Blend 1_E and 0.05 eV for Blend 2_E, which are less than the limiting condition. 

The XRD patterns of SN_E and PEO_E portrayed weak and broad characteristic peaks of succinonitrile and PEO, respectively, without the ionic salts’ peaks [62,64,65]. This indicated the molecular disorderedness of succinonitrile and a decrease in crystallinity of PEO along with a complete dissolution of ionic salts. On the contrary, Blends 1_E and 2_E had no characteristic peaks of ingredients, demonstrating the arrest of the glassy phase because of an interaction between PEO, succinonitrile, and ions [68,77,78]. These assertions can also be made using FT-IR spectroscopy. The FT-IR spectroscopy exhibited no significant change in modes of ionic salts and acetonitrile in ACN_E, revealing the least solvent-solute interaction. SN_E showed a similar scenario, however with an SN-bpy ligand interaction. PEO_E experienced a significant change in modes of ionic salts and PEO, revealing a conformational change of PEO by the large-sized ions. In contrast, the SN-PEO blend-based electrolytes, Blends 1_E and 2_E observed no significant change in modes, except at 777 and 1437 cm^−1^ for the SN-bpy ligand interaction and the ν_CH2_ modes. The matrix/solvent-salts interaction can be put in an order as: ACN_E < SN_E < Blends 1_E & 2_E << PEO_E. It is also worth mentioning that the FT-IR spectra did not show the stability of the electrolytes via the hydrogen interaction with the nitrile group. 

The transmittance spectra had the following order: Blend 2_E > Blend 1_E >> PEO_E >> ACN-E >> SN_E (=0%) in the UV-A region and ACN-E ≈ Blend 2_E (~100%) > SN_E(L) > Blend 1_E > PEO_E >> SN_E(S) in the visible region. These electrolytes were transparent in the near-infrared region too. This showed the transparency of Blend 2_E in a wide wavelength range, which makes it superior to ACN_E and $I^{-}/I_{3}^{-}$ redox couple redox mediators. The high level of transparency makes the blend-based electrolyte suitable for various types of solar cells, such as the Gratzel cells, back-illuminated DSSCs, and tandem solar cells [2,3,4,5,6,89]. Also, nearly 100% of transparency for this electrolyte revealed its glassy nature. The same was observed by the polarized optical microscopy too.

The DSC curve showed *T*_m_ peak at ~63.8 °C for PEO_E (~65.7 °C for PEO), ~47 °C for SN_E (~57.7 °C for SN), ~6 °C for Blend 1_E, and ~4 °C for Blend 2_E (~30.1 °C for PEO-SN blend). The area under the *T*_m_ peak corresponds to the heat of enthalpy of electrolyte, which had the following order: PEO_E >> SN_E >> Blend 1_E ≈ Blend 2_E (≈0). A decrease in *T*_m_-value and/ or the area indicated a decrease in crystallinity. Thus, PEO_E had a high level of crystallinity, and Blends 1_E and 2_E had a glassy nature. The TGA curves showed the thermal stability, up to ~75 °C for SN_E, ~200 °C for PEO_E, and ~125 °C for Blends 1_E and 2_E, which are similar to those of pure matrices [62]. 

## 5. Conclusions

We synthesized new Co^2+^/Co^3+^ solid redox mediators, [(1−*x*)SN: *x*PEO]-LiTFSI-Co(bpy)_3_(TFSI)_2_-Co(bpy)_3_(TFSI)_3_ with *x* equals to 0 (SN_E), 0.5 (Blends 1_E and 2_E), and 1 (PEO_E) in weight fraction. The electrolyte with SN was prepared identically just by replacing the ACN of the liquid redox mediator (ACN_E). The electrolytes exhibited σ_25__°C_-value in the following order, ACN_E (1.7 × 10^−2^ S cm^−1^) > SN_E (2.1 × 10^−3^ S cm^−1^) > Blend 2_E (7.2 × 10^−4^ S cm^−1^) > Blend 1_E (4.3 × 10^−4^ S cm^−1^) >> PEO_E (9.7 × 10^−7^ S cm^−1^). The log σ−*T*^−1^ study showed Arrhenius behavior for SN_E and PEO_E similar to ACN_E, and VTF behavior for Blends 1_E and 2_E. Only Blend-based solid polymer electrolytes showed activation energy of less than 0.3 eV, a high level of transparency in UV-A, visible, and IR regions, and thermal stability up to 125 °C, which are the basic requirements for the DSSC application in the Gulf region. This electrolyte is also suitable for tandem solar cell application. 

## Figures and Tables

**Table 1:** Composition of liquid and solid redox mediators.

*x* (Electrolyte)	EO/Li^+^	PEO (g)	ACN/ SN (g)	LiTFSI (g)	DN-C13 (g)	DN-C14 (g)
(ACN_E)	-	-	0.4425	0.0162	0.1531	0.0462
0 (SN_E)	-	-	0.5600	0.0162	0.1531	0.0462
0.5 (Blend 1_E)	113	0.2800	0.2800	0.0162	0.1531	0.0462
0.5 (Blend 2_E)	226	0.5600	0.5600	0.0162	0.1531	0.0462
1 (PEO_E)	226	0.5600	-	0.0162	0.1531	0.0462

**Table 2:** Observed vibrational frequencies (in cm^−1^) of the redox mediators, ACN_E, SN_E, PEO_E, and Blends 1_E & 2_E along with those of solvent/matrices and ionic salts.

ACN ^†^	ACN_E	SN	SN_E	Blend	Blend 1_E	Blend 2_E	PEO	PEO_E	Li Salt	Co Salt	Assignments ^‡^
		481s	479m			478w					δ_CCC_
	515m		514m		513m	514m		513m	513m	514m	
	571m		571m		571m	571m		570m	571s	571s	
	602sh	604s	602m	604m	601m	602m		601m	602m	601m	δ_CCC_
	620s		618s		618m	618m		617m	617m	616m	
	653m		653m		652w	652w		652w	654m	656m	
753m	739m		738m		738w	738w		738m	739m	740m	ν_a,C-CN_, ν_s,SNS_
	770s	762s	769s	762m	777m	778m		780m		767s	δ_CH2_, ring
	789m		789m		786sh	786sh		786sh	789m	789m	ν_a,SNS_
		819s	818m	819w							ν_C-CN_
				846m	849m	847m	843s	843m			ρ_a,CH2_, ν_CO_
919s	918m	918s	918m	918sh	sh	sh					ν_s,C-CN_
		963s	963s	953s	954m	953s	963s	961s			ρ_a,CH2_, t_CH2_, ν_C-CN_
		1002s	1002m	1002m	1003w	1002w					ρ_CH2_
1039s	1039sh										ρ_a,CH3_
	1061s		1059s	sh	1060sh	1061sh	1061m	1061sh	1059s	1057s	ν_a,COC_, ρ_a,CH2_, ν_a,SNS_
				1105s	1107s	1106s	1109s	1112s			ν_s,COC_
	1138m		1136s		1134sh	1134sh	1149s	1134s	1136s	1133s	ν_CC_, ν_s,SO2_
	1189s	1199m	1197s	1196w	1195s	1196s		1193s	1197s	1196s	t_CH2_, ν_a,CF3_
	1227sh	1233s	1228sh	sh	1227w	1228w			1228m	1229sh	t_CH2_, i.p.ring
				1251m	1250w	1250w	1242m	1242m			t_a,CH2_
				1299m	1301w	1301w	1280m	1281m			t_a,CH2_, t_s,CH2_
		1337s	1337sh	sh	1334sh	1334sh			1333sh	1331sh	ω_CH2_, ν_a,SO2_
	1350s		1353s	1350m	1353s	1353s	1342s	1349s	1353s	1351s	ω_a,CH2_, ν_a,SO2_
1374s	1376m										ω_s,CH2_
		1426s	1426s	1426s	1427w	1427m		1444w			δ_CH2_
1443s	1443m		1443m	1453w	1437w	sh	1454m	1454m		1453m	δ_a,CH2_, ring
	1474m		1474m	1469w	1472w	1472w	1467m	1468m		1470m	δ_a,CH2_, ring
2252s	2255s	2254s	2254s	2251s	2253m	2253s					ν_s,C__≡__N_
2293s	2293w			2875s	2876s	2877s	2861sh	2872sh			ν_s,CH2_
				2899s	2904s	2907s	2889s	2894s			ν_a,CH2_
2942m	2944w	2952s	2951s	2943sh	2944sh	2946sh					ν_s,CH2_
3001m	3004w	2989s	2989s	2975sh	2979sh	2981sh					ν_a,CH2_

^†^ Relative intensity notations: w, weak; m, medium; s, strong; and sh, shoulder. ^‡^ Assignment notations: ν, stretching; δ, bending; ω, wagging; t, twisting; ρ, rocking; s, symmetric; a, asymmetric; i.p., in-plane; and o.p., out-of-plane.

## Data Availability

Data is available up on request from the corresponding author.
